# Rapid One-Tube RPA-CRISPR/Cas12 Detection Platform for Methicillin-Resistant *Staphylococcus aureus*

**DOI:** 10.3390/diagnostics12040829

**Published:** 2022-03-28

**Authors:** Yanan Li, Zhonglin Shi, Anzhong Hu, Junsheng Cui, Ke Yang, Yong Liu, Guoqing Deng, Cancan Zhu, Ling Zhu

**Affiliations:** 1 Hefei Institutes of Physical Science, Chinese Academy of Sciences, Hefei 230031, China; yananl@mail.ustc.edu.cn (Y.L.); musen_L@163.com (Z.S.); anzhonghu620@163.com (A.H.); cjs@rntek.cas.cn (J.C.); keyang@aiofm.ac.cn (K.Y.); liuyong@aiofm.ac.cn (Y.L.); dgq@aiofm.ac.cn (G.D.); 2 Science Island Branch of Graduate School, University of Science and Technology of China, Hefei 230026, China; 3 Institute of Physical Science and Information Technology, Anhui University, Hefei 230601, China

**Keywords:** methicillin-resistant *Staphylococcus aureus*, *mecA* gene, RPA-CRISPR/Cas12a, one-tube, POCT

## Abstract

Methicillin-resistant *Staphylococcus aureus* (MRSA) is a severe health threat causing high-level morbidity and mortality in health care environments and in community settings. Though existing diagnostic methods, including PCR and culture-based methods, are routinely used in clinical practice, they are not appropriate for rapid point-of-care testing (POCT). Recently, since the development of the CRISPR/Cas technology, new possibilities for rapid point-of-care detection have emerged. In this study, we developed a rapid, accurate, and contamination-free platform for MRSA detection by integrating recombinase polymerase amplification (RPA) with the Cas12 system into one tube. Using this approach, visual MRSA detection could be achieved in 20 min. Based on the one-tube RPA-CRISPR/Cas12a platform, the assay results are visualized by lateral flow test strips (LFS) and fluorescent-based methods, including real-time and end-point fluorescence. This platform allows specific MRSA detection with a sensitivity of 10 copies for the fluorescence method and a range of 10–100 copies for the LFS. The results of 23 samples from clinical MRSA isolates showed that the coincidence rate was 100% and 95.7% of the fluorescence method and LFS, respectively, compared to qPCR. In conclusion, the one-tube RPA-CRISPR/Cas12a platform is an effective method for MRSA detection with significant potential in future practical POCT applications.

## 1. Introduction

Antimicrobial resistance (AMR) is a global health concern that impacts the treatment of common infectious diseases [1]. The World Health Organization has proclaimed that AMR is among the top ten public health threats worldwide. Moreover, the advent of modern medicine has led to the emergence of many antibiotic-resistant bacteria. One such severe threat is drug-resistant *Staphylococcus aureus*. *S. aureus* is a spore-less bacteria prevalent in nature and can survive a wide range of harsh environmental conditions [2,3]. It can cause a wide range of infections, usually involving the skin, soft tissues, bones, joints, and associated with indwelling catheters or prosthetic devices [4,5]. Initially described in 1961, methicillin-resistant *S. aureus* (MRSA) produces altered penicillin-binding protein (PBP2a) encoded by *mecA* gene, which reduces the affinity for β-lactam antibiotics, resulting in resistance to all penicillins, cephalosporins, and carbapenems, thereby rendering β-lactam armamentarium clinically ineffective [6,7]. Methicillin resistance in *S. aureus* is problematic for the success of antibiotic therapy, prolonging hospitalization, and increasing the risk of morbidity and mortality [8,9]. Since the MRSA expansion, infections have not been limited to major hospital centers and healthcare systems. Community-acquired MRSA has also emerged, affecting young people without medical contact, resulting in purulent skin infections or pneumonia [10,11]. Therefore, to prevent the spread of MRSA and instruct clinicians on medication selection, MRSA testing should not only be rapid and sensitive, but also performed at the point of care [12]. Currently, the MRSA detection methods mainly include microbiological culture and real-time PCR [13]. Despite meeting the criteria for MRSA screening standard, the culture-based method is not appropriate for rapid screening because it is time consuming and generates excessive amounts of medical waste [14]. While commercial PCR kits targeting the *mecA* gene for MRSA detection have been evaluated in several prospective clinical research [15,16]. However, due to the time-consuming procedure and the need for expensive equipment and professional personnel, this method is unsuitable for point-of-care. Therefore, a rapid, convenient, and low-cost technique for detecting MRSA should indeed be investigated.

Recently, the advent of CRISPR technology provided a promising and innovative solution for rapid diagnostics [17]. Cas12 is highly valued for its targeted recognition and collateral cleavage activities on DNA among the Cas proteins [18]. By designing CrRNAs for different target genes, the Cas12’s non-specific DNase activity can be specifically activated, enabling it to cleave the ssDNA Reporter for detection purposes [19,20]. At present, CRISPR/Cas12 is frequently used in combination with isothermal amplification techniques, such as recombinase polymerase amplification (RPA), for sensitivity-improving detection [21]. Considering that CRISPR and RPA both compete for targets and interfere with each other, the two systems cannot be combined directly [22]. CRISPR and RPA can be combined in two ways: adding the reagents in steps and waiting for the RPA to react before adding the CRISPR reagents, which is prone to aerosol contamination; or reducing the concentration of the CRISPR and RPA reagents, which weakens the mutual interference, but it is time-consuming, typically taking 90–120 min [23,24].

**Figure 1:**
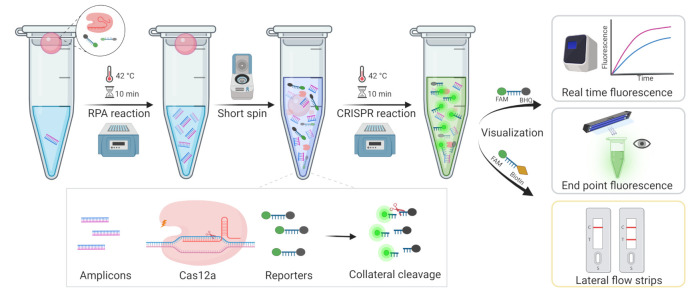
The workflow of the overall process for MRSA rapid detection based one-tube RPA-CRISPR/Cas12a assay within 20 min.

In this study, we developed an MRSA diagnostic system-based one-tube CRISPR/Cas12a method, which employs another strategy of adding RPA to the bottom of the tube and the CRISPR system to the cap, then mixing by spinning after the RPA reaction, temporarily separating the two systems. In this case, to visualize the results, fluorescence, including real-time fluorescence and endpoint fluorescence, and lateral flow strips (LFS) were used, respectively (Figure 1). This method could not only avoid aerosol contamination, but also reduce detection time, resulting in rapid, highly sensitive, and inexpensive detection with the potential to be utilized in point-of-care tests (POCT).

## 2. Materials and Methods

### 2.1. CrRNA Synthesis and Purification

Oligo DNA containing T7 promoter and complementary CrRNA sequences to transcribe CrRNA in vitro was synthesized by Sangon Biotech (Shanghai, China).The oligo DNA was annealed with T7-ALL oligonucleotide by performing at 90 ${}^{\circ }$C for 5 min, followed by slowly cooling the reaction to room temperature. Then, the CrRNA was synthesized by incubating at 37 ${}^{\circ }$C for 4 h with TranscriptAid T7 High Yield Transcription Kit (ThermoFisher Scientific, Waltham, MA, USA). Template DNA should be removed with DNase I digestion directly after the transcription reaction. TRIzol™ LS Reagent (ThermoFisher Scientific, Waltham, MA, USA) was employed to purification the RNA transcripts. Finally, CrRNA was asserted by 2% denaturing agarose gel electrophoresis and Nanodrop spectrophotometer (ThermoFisher Scientific, Waltham, MA, USA) and stored at −20 ${}^{\circ }$C.

### 2.2. Polymerase Chain Reaction (PCR) and Cas12a Fluorescence Detection

Partial sequences of MRSA *mecA* gene were synthesized by Sangon Biotech and cloned into pUC57 vectors, and transformed into *E. coli* DH5α. Recombinant pUC57 plasmid DNA containing the mecA gene was extracted by Plasmid Small Extraction Kit (Tiangen, Beijing, China). Then, we used M13F and M13R as primers and the extracted plasmid DNA as a template to amplify the *mecA* gene on Biometra Tone-PCR Thermal Cycler (Analytik Jena, Jena, Germany.) The PCR procedure follows the instructions of Premix Taq (Takara Bio Inc., Beijing, China): 30 cycles by 98 ${}^{\circ }$C for 10 s, followed by 52 ${}^{\circ }$C for the 30 s, and 72 ${}^{\circ }$C for 1 min. Finally, the PCR products were analyzed by 1% agarose gel electrophoresis. The Cas12a fluorescence detection experiment was carried out using EnGen^®^ LbaCas12a (NEB, Ipswich, MA, USA) and above purified CrRNAs. For each reaction, 1 μL of LbaCas12a (1 μM) preassembled with 100 ng of CrRNA and 0.25 μL RNA inhibitor. Then, 1 μL ssDNA FQ Reporter and 1.5 μL of 10× NEBuffer 2.1 were mixed; finally, 3 μL above PCR products were added and immediately incubated at 42 ${}^{\circ }$C on a LightCycler 96 system (Roche, Basel, Switzerland) to measure the Fluorescence kinetics.

### 2.3. One Tube RPA-Cas12a Detection Platform

The one-tube RPA-Cas12a detection platform combines RPA amplification and Cas12a detection. The RPA amplification was performed at the bottom of the tube according to the TwistAmp Liquid Basic kit (TwistDx, Maidenhead, UK) with modifications. For a 25 μL of RPA reaction contains a premix of 10 μL of 2× Reaction Buffer, 1 μL of dNTPs, 2 μL of 10× E-mix and 1 μL each of RPA primer F and R; 1 μL of 20× Core Reaction mix, 2 μL of template DNA; 2 μL of MgOAc was added at the end. In addition, the CRISPR/Cas12a system was put at the tube lid, which included 1 μL of LbaCas12a (1 μM), 1 μL of CrRNA (2 μM) and 0.25 μL RNA inhibitor, 1 μL ssDNA FQ Reporter and 2 μL of 10× NEBuffer 2.1. The tubes should be covered gently, placed on a thermostat and incubated at 42 ${}^{\circ }$C for 10 min to perform the PRA amplification reaction. At that time, the Cas12a system was briefly spun down and mixed with the RPA amplicons, and they were immediately placed on the LightCycler 96 system and incubated at 42 ${}^{\circ }$C for real-time fluorescence acquisition. To monitor the real-time progress of the reactions, the fluorescence intensity was recorded every 1 min.

### 2.4. Visual End-Point Detection with a UV Light Illuminator

The one-tube RPA-Cas12a reactions were carried out using the same procedures as previously described. Following spin-down of the Cas12a system, they were immediately re-incubated at 42 ${}^{\circ }$C for 10–15 min. For visual detection, the tubes were illuminated with Blue Light Gel Imager (Songon Biotech, Shanghai, China) and photographed using a cell phone camera for analysis.

### 2.5. Lateral Flow Strip Assay

The RPA-CRISPR/Cas12a-based Lateral flow strip assays were performed using the FB Reporter whose ssDNA are modified with Biotin and FAM at two ends. In the LFSA essay, the RPA amplification method was followed the above described. The Cas12a system contained 1 μM of LbaCas12a, 2 μM CrRNA and 0.25 μL RNA inhibitor, 5 μM ssDNA FB Reporter and 3 μL of 10× NEBuffer 2.1. While Cas12a reagents were mixed with RPA amplicons, the mixture requires incubating in the thermostat at 42 ${}^{\circ }$C for 10–20 min. Then, the reaction mixture was upped to 100 μL by adding ddH${}_{2}$O and applied to a lateral flow strip (JY0301, Tiosbio, Nanjing, China). The results were assessed 5–10 min after they were applied.

### 2.6. Bacterial Strains and Clinical MRSA Samples Preparation

In this study, the standard strains methicillin-resistant *Staphylococcus aureus* (ATCC-43300), *Staphylococcus aureus* (ATCC-29213), *Klebsiella pneumoniae* (ATCC-700603), *Escherichia coli* (ATCC-25922) were provided by the American Type Culture Collection (ATCC); *Acinetobacter baumannii* (BIO-53272), *Pseudomonas aeruginosa* (BIO-109004) were provided by BIOBW (China). Additionally, A total of 23 MRSA isolates were isolated from clinical samples provided by the clinical laboratory of the First Affiliated Hospital of Anhui Medical University. According to the manufacturer’s instructions, the genomic DNA of bacterial strains was extracted with MagPure Bacterial DNA KF Kit (Magen, Guangzhou, China) and then validated by 1% agarose gel electrophoresis.

### 2.7. Real-Time PCR Detection and Standard Curve

The TaqMan real-time PCR detection of the *mecA* gene was carried out using a LightCycler 96 system (Roche, Basel, Switzerland) according to SensiFAST™ Probe No-ROX Kit (Bioline, Luckenwalde, Germany) recommended procedure described with the following modifications. Briefly, single-tube PCR were prepared to contain 10 μL of 2× SensiFAST Probe No-ROX Mix, 1 μL of primer F, 1 μL of primer R, 0.5 μL of TaqMan probe, 2 μL of DNA sample, and 3.5 μL of ddH${}_{2}$O. The amplification conditions used were an initial denaturation step of 95 ${}^{\circ }$C for 3 min, followed by 40 cycles of 95 ${}^{\circ }$C for 10 s, and 58 ${}^{\circ }$C for the 30 s, finally cooling at 37 ${}^{\circ }$C. Fluorescence information was collected at the 58 ${}^{\circ }$C annealing extension per cycle. The standard curve for quantification was prepared by diluting the pUC57 plasmid DNA of *mecA* into 10${}^{6}$, 10${}^{5}$, 10${}^{4}$, 10${}^{3}$, 10${}^{2}$, 10${}^{1}$ copies/per reaction, yielding a highly significant relationship $\left(R^{2}\geq 0.99\right)$, and slopes were within the acceptance criterion (between −3.1 and −3.6).

### 2.8. Statistical Analysis

All results generated from at least three independent experiments. Statistical analyses and graphing were performed using GraphPad Prism 8.

## 3. Results

### 3.1. Development of One-Tube RPA-CRISPR/Cas12a Assay for MRSA Detection

**Figure 2:**
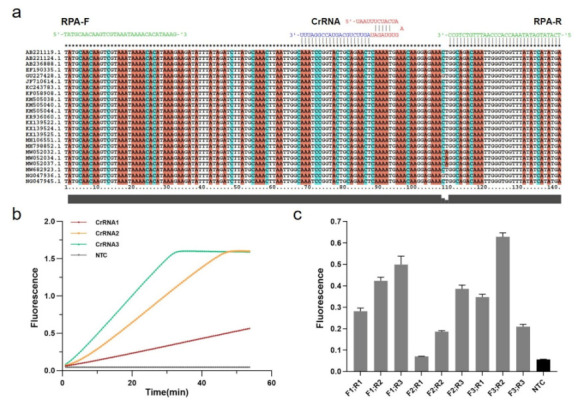
Establishment of RPA-CRISPR/Cas12a system. (**a**) Relatively conserved sequence of *mecA* gene of MRSA shows target and crRNA sequences on the polymerase coding region. (**b**) Screening for CrRNAs. Real-time fluorescence curves based on PCR-CRISPR/Cas12a on different 3 CrRNAs to detect efficiency. NTC: no template control. (**c**) Screening of RPA primers. Fluorescence values based on RPA-CRISPR detection at different RPA primers. Gray: Postive; Black: Negative.

The Cas12a targeting sequence was designed based on 23 *mecA* gene-related *S. aureus* strains acquired from GenBank (Figure 2a). The MUSCLE algorithm in MEGA6 was used to recognize relatively conserved sequences by multiple sequence alignment. Cas12a protospacer adjacent motif (5${}^{\prime }$-TTTV) on the conserved sequence region was screened to design guide RNAs sequence (gRNAs) complementary to the target site, and the CRISPR RNAs (CrRNAs) including gRNAs and conserved stem-loop sequence were designed for subsequent MRSA Cas12a detection assays (Table S1).

Three CrRNAs were designed and synthesized in vitro by the T7 transcription essay, which were examined by denaturing agarose gel electrophoresis (Figure S1A). Following the purification, the concentrations were measured by NanoDrop and diluted with DEPC H${}_{2}$O to a final concentration of approximately 100 ng/μL (Figure S1B). To achieve the best detection result in Cas12a-based diagnostics, these CrRNAs should be tested. Thus, we used PCR to amplify the recombinant pUC57 plasmid containing a partial mecA gene (Figure S2), and then the partial amplicons were detected by CRISPR fluorescence detection experiment with different CrRNAs. According to the real-time fluorescence acquisition on LC96, we discovered that, while the final fluorescence intensities were the same, the activities of three CrRNAs differed, with CrRNA3 demonstrating the highest efficiency due to the sharpest slope (Figure 2b).

In addition to the Cas12a system, it is also crucial to establish the RPA system in the One-tube RPA-CRISPR/Cas12a-based assay for MRSA detection. Three pairs of conserved RPA primers (30–33 bp) were designed with Oligo7 based on the identified sequences at both ends of CrRNA3 for subsequent screening (Table S1). Using the TwistAmp Liquid Basic kit, RPA amplification with different primer pair combinations was carried out for real-time fluorescence detection, combined with the Cas12a assay (Figure S3). Moreover, experiments were performed in triplicate (mean ± standard deviation), and the primer pair F3 and R2 with the highest fluorescence represents the optimum (Figure 2c). Consequently, we conducted subsequent experiments using the CrRNA3 and RPA primers F3 and R2.

### 3.2. Optimization of Experimental Conditions

**Figure 3:**
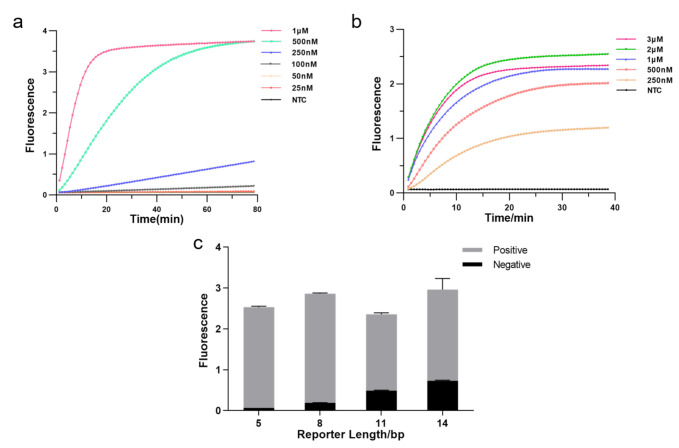
Optimizing the RPA-CRISPR reaction system. (**a**) Real-time fluorescence curves based RPA-CRISPR/Cas12a at different Cas12a protein concentrations. NTC: no template control. (**b**) Real-time fluorescence curves based RPA-CRISPR/Cas12a at different CrRNA concentrations. (**c**) Optimization of the FQ reporter length for the CRISPR detection. Various lengths (5, 8, 11, 14 bp) of FQ ssDNA reporter were tested to avoid false-positive results (*n* = 3, error bars showed mean with SD).

The CRISPR system consists of three essential components: Cas12a enzyme, CrRNA, and reporter, and we optimized each element to enhance detection efficiency. Firstly, we tested the effect of different Cas12a enzyme concentrations on the experiments by real-time fluorescence. The result showed that fluorescence reached a plateau more quickly as the enzyme concentration increased, allowing the detection to be more effective. Hence, we applied 1 μM as Cas12a concentration (Figure 3a). Then, we explored the optimal CrRNA concentration. The result illustrated that a higher concentration of CrRNA was used, a shorter time was taken to reach saturated fluorescence intensity and higher fluorescence intensity. The detection efficiency was better when the CrRNA concentration reached 2 μM. Therefore, we chose a CrRNA concentration of 2 μM for the subsequent assay (Figure 3b). We further optimized the length of FQ reporters since the reporter is crucial in fluorescence analysis. As illustrated in Figure 3c, the fluorescence intensity increased with extending the length of the FQ reporter, but background fluorescence also increased; fluorescence intensity decreased as reporter length increased beyond 8 bp, but background fluorescence still rose. In our subsequent experiments, we employed the 5 bp FQ reporter to avoid the impact of background fluorescence causing false positives.

### 3.3. Sensitivity of the One-Tube RPA-CRISPR/Cas12a Detection

**Figure 4:**
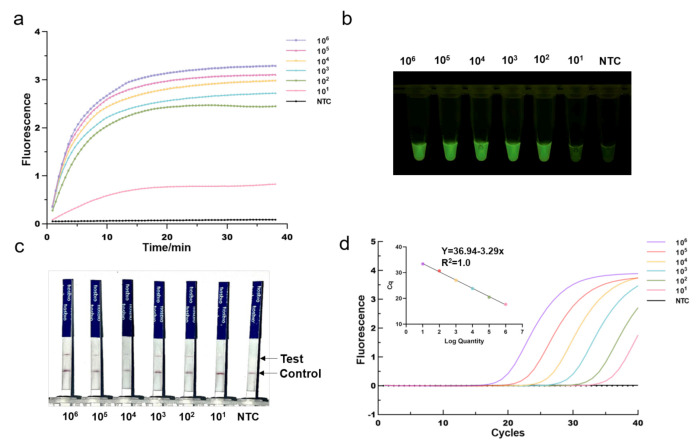
Determination of the sensitivity of RPA-CRISPR/Cas12a assays. Different concentrations of MRSA Plasmids (10${}^{1}$ to 10${}^{6}$ copies per reaction) were used test. (**a**) Real-time fluorescence RPA-CRISPR/Cas12a detection of different MRSA dilution. NTC: no template control. (**b**) Endpoint fluorescence/visual detection to MRSA plasmid dilution. (**c**) LFS-based RPA-CRISPR/Cas12a detection assay of MRSA plasmid dilution. Except for the blurred sample 10 copies/reaction test lines, all samples had clear test lines. (**d**) qPCR amplification curves and standard curves for MRSA plasmid dilution.

To investigate its sensitivity for MRSA DNA detection, we first applied the optimized One-tube RPA-CRISPR/Cas12a assay to detect serial diluted MRSA plasmid DNA templates with mecA gene fragments (from 10${}^{1}$ to 10${}^{6}$ copies/per reaction). We found that the fluorescence intensity gradually declined as the concentration of diluted plasmid DNA decreased. As shown in Figure 4a, the RPA-CRISPR could detect as low as 10 copies of MRSA plasmid DNA in real-time detection in 40 min. Despite being incubated for 40 min, the one-tube RPA-CRISPR system was able to identify 10 copies of MRSA DNA after just 10 min of incubation based on end-point fluorescence, as illustrated in Figure 4b. For comparison, qPCR was conducted in the same diluted plasmid (Figure 4d). The *mecA* gene, determined by RPA-CRISPR and qPCR, always exhibited a consistent positive signal in all the tested samples. Therefore, RPA-CRISPR/Cas12a based on fluorescence had comparable sensitivity for detecting the MRSA to qPCR. In addition, we also evaluated the sensitivity of RPA-CRISPR/Cas12a based on lateral flow strips (Figure 4c). The results showed that the sample at 100 copies and above, the test bands are clearly visible, whereas at 10 copies, they exhibited blurred test bands, indicating that RPA-CRISPR/Cas12a based LFS test has a detection range of 10–100 copies.

### 3.4. Specificity of the One-Tube RPA-CRISPR/Cas12a Detection

**Figure 5:**
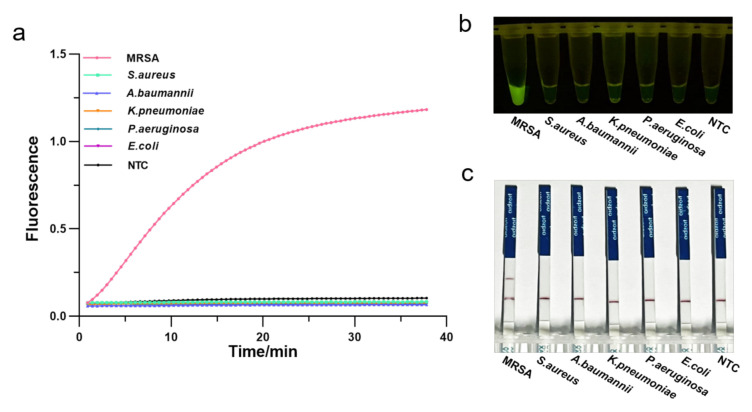
Determination of the specificity of one-tube RPA-CRISPR/Cas12a detection of MRSA using 5 common pathogenic bacteria. (**a**) Real-time fluorescence intensity curves of the RPA-CRISPR/Cas12a assays for MRSA and different bacteria strains. NTC: no template control. (**b**) Endpoint visualization fluorescence-based one-tube RPA-CRISPR/Cas12a detection of MRSA and 5 different bacteria strains. A strong fluorescent signal was observed with the naked eye only when MRSA sample were added. (**c**) Specificity test of the RPA-CRISPR/Cas12a LFS detection assay. Only the MRSA sample showed a clear test line.

To assess the specificity of the RPA-CRISPR/Cas12a assay, we employed several pathogenic bacteria commonly found in the hospitals, such as methicillin-resistant *Staphylococcus aureus*, susceptible *Staphylococcus aureus*, *Klebsiella pneumoniae*, *Escherichia coli*, *Acinetobacter baumannii*, *Pseudomonas aeruginosa*. Genomic DNA of these strains was extracted and analyzed by agarose gel electrophoresis (Figure S4), followed by the one-tube RPA-CRISPR/Cas12a detection. As a result, only samples of MRSA exhibited a strong fluorescence signal in real-time detection without cross-reactivity with other pathogenic bacteria (Figure 5a). Consistently, the visual detection by a UV light illuminator showed that RPA-CRISPR/Cas12a could specifically distinguish the MRSA (Figure 5b). Furthermore, LFS analysis revealed the MRAS test to be specific, with a distinct test line appearing only on the LFS where MRSA was added (Figure 5c). Therefore, the RPA-CRISPR/Cas12 method could specifically identify only the MRSA.

### 3.5. Validation of One-Tube RPA-CRISPR/Cas12a MRSA Detection Assay for Clinical Isolates

**Figure 6:**
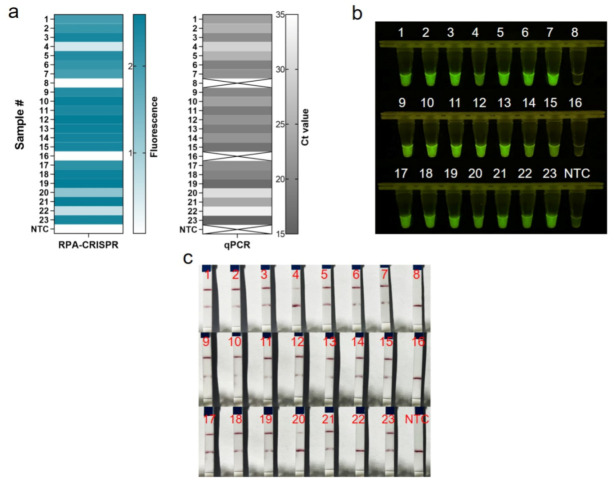
Evaluation of RPA-CRISPR/Cas12a assay for MRSA with 23 clinical isolates. (**a**) Real-time fluorescence-based one-tube RPA-CRISPR/Cas12a detection of MRSA and selection of fluorescence of CRISPR reactions for 10 min for heat map, validated by qPCR in parallel. Samples 8 and 16 are negative, the rest are positive. ×: indicates a negative sample. NTC: no template control. (**b**) Endpoint visualization fluorescence-based one-tube RPA-CRISPR/Cas12a detection of MRSA. The only samples that do not show fluorescence signals are samples 8 and 16. (**c**) Detection of MRSA in clinical isolates with the RPA-CRISPR/Cas12a LFS assay. Test lines are visible on all samples except samples 8, 16 and 22, which do not show test lines.

We assessed the performance of One-tube RPA-CRISPR/Cas12a to diagnose MRSA in clinical samples. In this study, 23 clinical isolates of MRSA were first tested by qPCR diagnostic testing, which served as the gold standard test for the clinical determination of MRSA. For the 21 positive samples, the cycle threshold (Ct) values of the qPCR ranged from 15.46 to 33.18, and the copies number could be calculated ranged from 3451,389.08 to 14.19 (Table S2). Clinical samples were assayed using the one-tube RPA-CRISPR/Cas12a system by using the fluorescent method and LFS techniques. Based on real-time fluorescent analysis, RPA-CRISPR was able to detect 21 positive samples and two negative samples, as determined by the qPCR assay (Figure 6a). Additionally, the endpoint visualization fluorescence took only 10 min CRISPR incubation to detect the positive samples mentioned above, and the intensity trend of fluorescence was basically consistent with the qPCR results (Figure 6b). The results showed that the RPA-CRISPR-based LFS method for MRSA detection detected 20 positive samples and three negative samples, but sample #22 could not be detected due to insufficient copy number concentration (Figure 6c). All in all, the coincidence rate of the LFS-based RPA-CRISPR/Cas12 assay was 95.7%, while the coincidence rate of the fluorescence-based RPA-CRISPR/Cas12 method was 100% (Table 1).

## 4. Discussion

*Staphylococcus aureus* is spread worldwide as a bacterial pathogen, and it represents an increasing health risk [25]. MRSA, in particular, has emerged as a widespread cause of severe infection both in hospitals and in the communities [5]. The World Health Organization (WHO) estimates that MRSA accounts for 20% of all documented cases of *S. aureus* infections, although that figure may surpass 80% in some developing countries [26]. As such, there is an increasing need for rapid and accurate MRSA detection that can be deployed at the point of care in low-resource settings. In addition to the primarily popular culture-based susceptibility tests and nucleic acid-based *mecA* gene detection methods for MRSA rapid detection, membrane protein-based methods for PBP2a identification have been developed [27]. MRSA was detected by integrating dual aptamer technology, and CRISPR-Cas12a assisted rolling circle amplification (RCA) to identify the membrane protein shared by both MRSA and SA, as well as specific PBP2a [28]. Although the introduction of the CRISPR method has improved the sensitivity of MRAS detection, it is still not as reliable and accurate as of the mecA gene sequencing methods. Therefore, we tested MRSA by integrating RPA and CRISPR/Cas12a to detect the *mecA* gene.

Several CRISPR proteins have diagnostic potential: Cas12, Cas13, and Cas14, all of which have non-sequence-specific trans-nuclease activities triggered by binding to specific targets [20,29,30]. These proteins target different nucleic acids, Cas12 targets dsDNA, Cas13 targets ssRNA, and Cas14 targets ssDNA [31]. Among them, Cas13 can detect DNA, but a redundant step of transcribing the target into RNA is required, and the reporter used in Cas13 is ssRNA, which can lead to false positives and is challenging to preserve. For the Cas14-based DNA detection, modified primers and T7 exonuclease are required for generating ssDNA substrate from dsDNA amplicons [30]. The Cas12 protein, in contrast, does not require an additional step, and thus, is widely used in DNA detection due to its ability to recognize DNA targets directly [32]. To increase the sensitivity of the assay, isothermal amplification is usually introduced before CRISPR, such as LAMP and RPA [33]. Due to the complexity of LAMP design and the reaction temperature being higher than the CRISPR, around 65 ${}^{\circ }$C, it was more common to use RPA amplification [22,34]. Since both the RPA and CRISPR systems react at 37 ${}^{\circ }$C to 42 ${}^{\circ }$C, integrating the two is more likely to succeed. Nevertheless, since two systems react sequentially, the intermediate operation of opening the lid can create aerosols that can contaminate the entire environment. Consequently, we employed the one-tube RPA-CRISPR/Cas12 platform to accomplish rapid, sensitive, and contamination-free detection of MRSA.

To develop the RPA-CRISPR/Cas12 detection system, we set up and optimize the CRISPR system. CrRNA and Reporter are critical in the CRISPR/Cas12 assay. Since different CrRNAs are designed for detecting different targets, selecting the proper CrRNA is critical to developing an effective CRISPR/Cas12 detection system [35]. Due to the difficulty of predicting the activity of CrRNA presently, we designed several CrRNAs and tested experimentally to select the most effective. The result indicates that all three CrRNAs we designed could detect MRSA, but their efficiencies varied, so we selected the most efficient CrRNA3 for further analysis. In addition, because the Reporter directly influences the fluorescence signal, we examined how different lengths of a Reporter affect it. We found that increasing Reporter length resulted in a stronger basal fluorescence signal, which is consistent with the publication of Liu [32]. Possibly, the longer the ssDNA is, the more likely it is to undergo self-cleavage, and the further distance between FAM and BHQ1, the less likely BHQ1 will quench the fluorescence emitted by FAM. Based on the fact that an increase in basal fluorescence affects the determination of results at low concentrations and increases the risk of false positives, we have chosen the 5bp Reporter with the lowest basal fluorescence.

In this study, we employed three visualization methods to present the detection results of MRSA: real-time fluorescence, visible endpoint fluorescence, and lateral flow strips. The first two methods rely on the fluorescence method, employing the same reaction system and having a sensitivity of 10 copies almost comparable to PCR. Using the real-time fluorescence method has been very useful for setting up experimental systems. This method enables us to monitor the entire CRISPR reaction process, optimizing the entire system and determining the optimum reaction time. However, this method is not suitable for POCT in resource-poor areas because of the need for expensive real-time fluorescence acquisition equipment. On the contrary, the RPA-CRISPR/Cas12 based visible endpoint fluorescence method has more potential to apply in the point-of-care testing, and it has been extensively studied in other applications [36]. This method allows us to obtain results after 5 to 10 min of CRISPR incubation by illuminating the test tubes with an easily accessible UV lamp. The entire process can be accomplished in 15–20 min without contamination from opening the cap, and without expensive instruments, only a tiny centrifuge, a thermal incubator, and a UV light are required for the test. Furthermore, we also use LTS to present the test results. As shown in Figure 4c, the detection range of LTS is between 10 and 100 copies, which has been demonstrated in clinical MRSA isolates, detecting 77.73 copies for sample #20, but not 14.19 copies for sample #22 (Table S2). Thus, the LTS method has a lower detection sensitivity than the fluorescence method and introduces aerosol contamination during the detection process due to the opening of the tube cap. However, this method does not require additional devices to display the results, making it more convenient and low-cost. If it were integrated into a hermetic device in the future, there would be a great deal of application potential to the POCT [37].

## 5. Conclusions

In summary, we developed a rapid, sensitive, and contamination-free one-tube RPA-CRISPR/Cas12a system for detecting MRSA, which could present test results in two ways: fluorescence methods and lateral flow strips. Based on the results obtained, this system has demonstrated high specificity without any cross-reactivity among different bacteria strains and extremely high sensitivity. The fluorescence-based test has a detection sensitivity of 10 copies, which is comparable to qPCR; the lateral flow strip-based test has a detection sensitivity in the range of 10–100 copies. In addition, we validated this system with clinical MRSA isolates and confirmed its potential for clinical applicability. Thus, this system is simple and non-polluting, requires inexpensive instrumentation, takes only around 20 min to complete, and could potentially be applied to POCT in the future.

## Figures and Tables

**Table 1:** Clinical validation of one-tube RPA-CRISPR/Cas12 detection for the MRSA.

		qPCR		Coincidence Rate (CR)
		Positive	Negative	
RPA-Cas12a fluorescence-based	Positive	21	0	100%
	Negative	0	2	
	Total	21	2	
RPA-Cas12a lateral flow strip	Positive	20	0	95.7%
	Negative	1	2	
	Total	21	2	

Twenty-three clinical isolates were used to evaluate the RPA-CRISPR/Cas12 assay. Coincidence Rate (CR) = 100% × [(both positive + both negative)/total samples]; qPCR is the gold standard for MRSA.

## Data Availability

The data that support the findings of this study are available from the corresponding author upon a reasonable request.

## Supplementary Materials

The following supporting information can be downloaded at: https://www.mdpi.com/article/10.3390/diagnostics12040829/s1, Figure S1: Synthesis and purification of crRNAs, Figure S2: Electrophoresis analysis of PCR amplicons of MecA gene, Figure S3: Real-time fluorescence curve based on RPA-CRISPR/Cas12 for RPA primer screening, Figure S4: Electrophoretic analysis of several bacterial genomes after extraction, Table S1: Sequences used in this experiment, Table S2: The Ct values of qPCR for 23 samples and their copy numbers.
