# Supplementary material for: Rapid One-Tube RPA-CRISPR/Cas12 Detection Platform for Methicillin-Resistant Staphylococcus aureus
Source: Diagnostics (Basel). 2022 Mar 28;12(4):829. doi: 10.3390/diagnostics12040829 (PMC9028452; doi:10.3390/diagnostics12040829)
Supplement: Supplementary file 1 [file diagnostics-12-00829-s001.zip › diagnostics-1650742-supplementary.pdf]

Supplementary information

Figure S1 Synthesis and purification of crRNAs

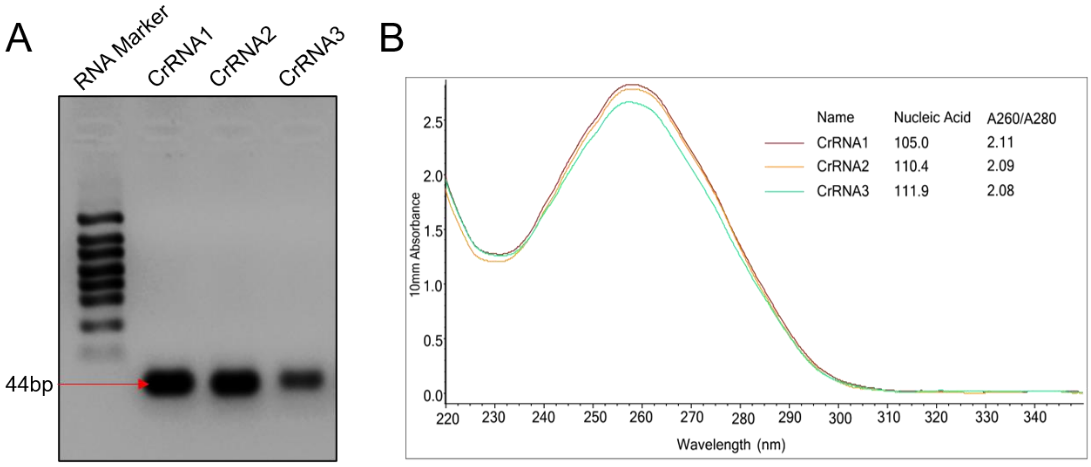

(A) Electrophoresis analysis of crRNA for MRSA from in vitro transcription. Lane 1 ,2 and 3 were crRNAs for S. aureus before purification. (B) Absorbance curves of purified and diluted CrRNAs.

Figure S2 Electrophoresis analysis of PCR amplicons of *MecA* gene

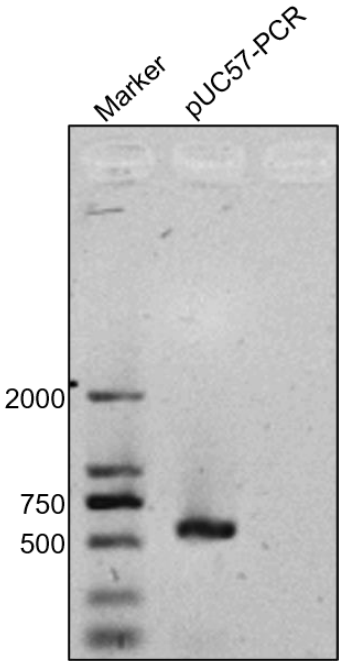

Figure S3 Real-time fluorescence curve based on RPA-CRISPR/Cas12 for RPA primer screening

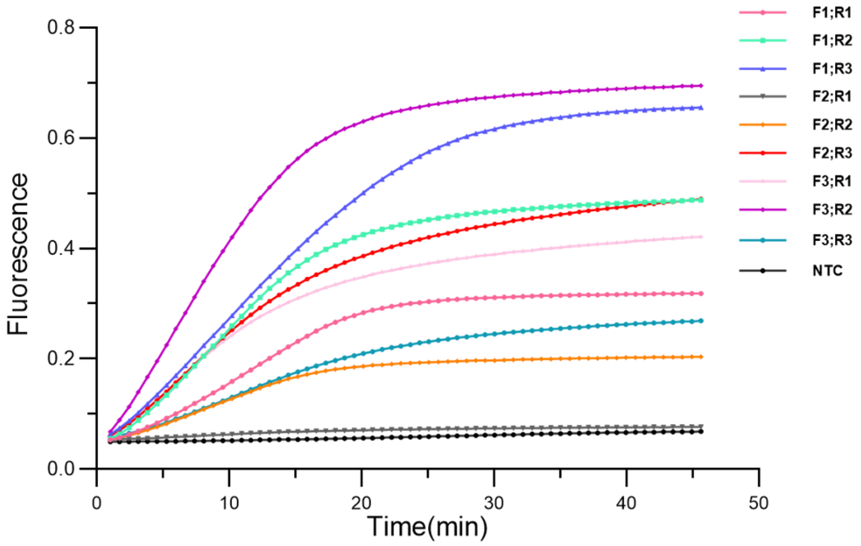

Figure S4 Electrophoretic analysis of several bacterial genomes after extraction

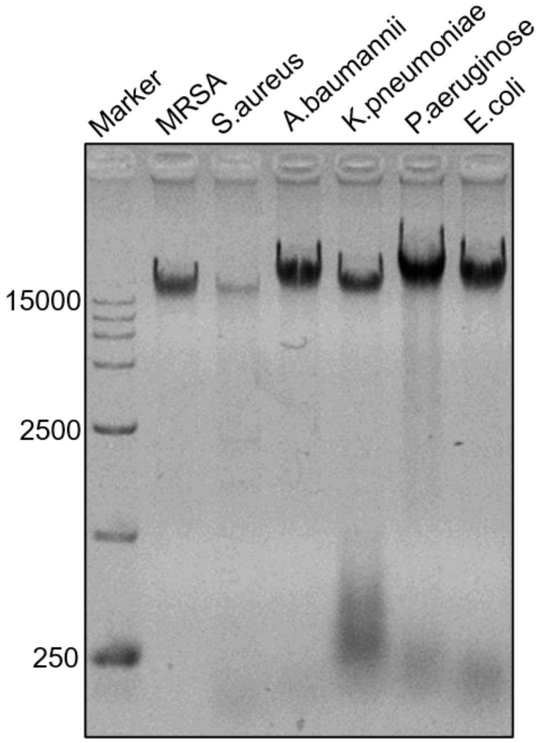

Table S1 Sequences used in this experiment

Primer Name	Sequence 5’-3’
T7-ALL	GAAATTAATACGACTCACTATAGGG
mecA-CrRNA1	UAAUUUCUACUAAGUGUAGAUUAGAUCUUAUGCAAACUUAA
T7- mecA-CrRNA1	TTAAGTTTGCATAAGATCTAATCTACACTTAGTAGAAATTA*CCCTATAGTGAGTCGTATTAATTTC*
mecA-CrRNA2	UAAUUUCUACUAAGUGUAGAUCATAAGATCTATAAATATCT
T7- mecACrRNA2	AGATATTTATAGATCTTATGATCTACACTTAGTAGAAATTA*CCCTATAGTGAGTCGTATTAATTTC*
mecA-CrRNA3	UAAUUUCUACUAAGUGUAGAUAGUUCUGCAGUACCGGAUUU
T7- mecA-CrRNA3	AAATCCGGTACTGCAGAACTATCTACACTTAGTAGAAATTA*CCCTATAGTGAGTCGTATTAATTTC*
mecA-RPA-F1	TCAATCTATTAACTGATGGTATGCAACAAGTCG
mecA-RPA-F2	TGATGGTATGCAACAAGTCGTAAATAAAACAC
mecA-RPA-F3	TATGCAACAAGTCGTAAATAAAACACATAAAG
mecA-RPA-R1	TCTTTATCATATGATATAAACCACCCAATTTG
mecA-RPA-R2	TCATATGATATAAACCACCCAATTTGTCTGCC
mecA-RPA-R3	TATGATATAAACCACCCAATTTGTCTGCCAG
FQ Reporter	5’-6-FAM-TTATT-BHQ1-3’
FB Reporter	5’-6-FAM-TTATTTTATTTTATT-BHQ1-3’
mecA PCR F	TGGATGAATATTTAAGTGATTTCGC
mecA PCR R	AATGGGACCAACATAACCTAATAGATG
mecA PCR Probe	AGAAAGTCGTAACTATCCTCTAGG
PUC57-M13R	CAGGAAACAGCTATGACC
PUC57-M13F	TGTAAAACGACGGCCAGT

Table S2 **The Ct values of qPCR for 23 samples and their copy numbers.**

Sample Number	Ct value	Copies
1	22.78	20562.22
2	25.28	3574.30
3	20.45	105021.11
4	29.34	208.52
5	25.44	3195.65
6	19.14	262695.65
7	21.1	66635.89
8	/	/
9	19.66	182556.87
10	23	17627.90
11	18.31	469604.80
12	21.4	54016.05
13	18.59	386034.52
14	21.51	50013.60
15	17.24	993025.70
16	/	/
17	20.87	78273.91
18	19.39	220528.66
19	16.42	1762790.12
20	30.75	77.73
21	24.03	8572.96
22	33.18	14.19
23	15.46	3451389.08
NTC	/	/
